# RSV Antibody Prophylaxis Needs for Extremely Preterm Infants in Their Second RSV Season

**DOI:** 10.1001/jamapediatrics.2026.0035

**Published:** 2026-03-09

**Authors:** Marina Viñeta Paramo, Allison Watts, Alfonso Solimano, Claire Seaton, Manish Sadarangani, Hind Sbihi, Pascal M. Lavoie

**Affiliations:** 1 Department of Pediatrics, University of British Columbia, Vancouver, British Columbia, Canada; 2 Epidemiology and Intelligence Services, BC Centre for Disease Control, Vancouver, British Columbia, Canada; 3 British Columbia Children’s Hospital Research Institute, Vancouver, British Columbia, Canada

## Abstract

This cohort study examines children in British Columbia, Canada, born between 2013 and 2024 to assess whether use of monoclonal antibodies for a second respiratory syncytial virus (RSV) season is warranted for extremely preterm infants.

Long-acting monoclonal antibodies (mAbs) have proven effectiveness against respiratory syncytial virus (RSV) hospitalizations in infants.^1^ For children who remain persistently at high risk, advisory committees recommend use of mAbs for a second season. However, premature children are excluded unless they have other underlying chronic medical conditions, such as bronchopulmonary dysplasia or severe immunodeficiency.^2,3^ Although a previous study examined RSV hospitalization rates by postnatal age among children born preterm,^4^ none provided sufficient gestational-age (GA) granularity to estimate risk specifically among infants born at less than 28 weeks’ gestation or beyond 12 months of postnatal age. This study reports RSV hospitalization rates at the population level, among preterm children born in British Columbia (BC), Canada, in their first and second RSV seasons.

## Methods

This population-based birth cohort study included all children live born in BC from April 1, 2013, to March 31, 2024, registered in the provincial health plan (>98% of births). Children for whom GA was missing were excluded. This study followed the STROBE reporting guideline. This study was a program evaluation approved by the Provincial Health Services Authority; ethics review was waived by the University of British Columbia Children’s and Women’s Research Ethics Board because the board determined the study to be categorized as program evaluation, which does not require review.

The primary outcome was RSV hospitalization based on *International Statistical Classification of Diseases and Related Problems, Tenth Revision, Canada* codes as most responsible or contributory diagnosis (J12.1, J20.5, J21.0, and B97.4). The methodology was described elsewhere, including the chronic medical condition classification.^5^ This outcome previously showed 80.1% sensitivity and 97.9% specificity capturing RSV test-positive hospital admissions in BC.^5^ Main exposure was GA at birth, stratified.

RSV hospitalization rates by postnatal age were estimated with a Poisson model and plotted using locally weighted scatterplot smoothing. Season-stratified rates were estimated using a Poisson generalized estimating equation model to account for repeated measures,^5^ by season (October to September, to capture residual postseason admissions) (eFigure in Supplement 1). Statistical precision was quantified using 95% CIs. All analyses were conducted using R statistical software, version 4.3.1.

## Results

Of 473 386 children, 443 221 (93.6%) were included (30 165 were missing data on GA and therefore were excluded). Overall, 4649 children (1.0%) experienced 4709 RSV hospitalizations before their 2-year birthday. RSV hospitalizations peaked before 3 months in full-term children. However, for children born at less than 28 weeks’ GA, RSV hospitalizations peaked between 15 and 17 months (Figure). There were more chronic medical conditions among premature children (Table).

**Figure.**
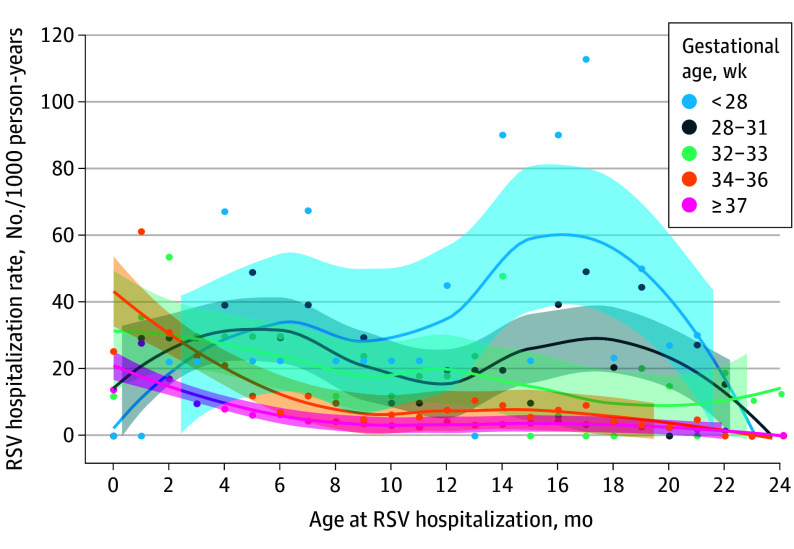
Line Graph Showing Respiratory Syncytial Virus (RSV) Hospitalization Rates by Postnatal Age and Prematurity Categories Points represent observed incidence rates per 1000 person-years by month of age. Lines show smoothed estimates using locally weighted scatterplot smoothing, with shaded areas indicating 95% CIs to quantify the statistical precision around the fitted mean.

**Table.** Season-Stratified RSV Hospitalization Rates by Gestational Age Category

Baseline characteristic	Gestational age category				
	≥37 wk (n = 397 883)	34-36 wk (n = 37 236)	32-33 wk (n = 4399)	28-31 wk (n = 2574)	<28 wk (n = 1129)
Sex, No. (%)					
Female	195 255 (49.1)	17 149 (46.1)	1918 (43.6)	1166 (45.3)	516 (45.7)
Male	202 627 (50.9)^a^	20 083 (64.3)^b^	2481 (56.4)	1408 (54.7)	613 (54.3)
Chronic medical conditions, No. (%)	17 707 (4.5)	4946 (13.3)	881 (20.0)	858 (33.3)	626 (55.4)
Maternal age, median (IQR), y	32.6 (28.7-36.2)	32.1 (28.7-35.4)	32.6 (29.0-36.2)	32.5 (28.9-36)	32.7 (28.9-36.3)
**RSV-LRI outcomes in first season**					
Hospital admissions, No. (%)	2567 (0.6)	499 (1.3)	72 (1.6)	64 (2.5)	16 (1.4)
PICU admissions, No. (% of RSV hospitalizations)	245 (9.6)	61 (12.0)	13 (18.0)	11 (18.0)	7 (47.0)
Adjusted hospital admission rate per 1000 PY (95% CI)^c^	6.95 (6.68-7.22)	13.54 (12.34-14.74)	15.79 (12.14-19.44)	22.55 (16.86-28.24)	12.98 (6.25-19.71)
**RSV-LRI outcomes in second season**					
Hospital admissions, No. (%)	937 (0.2)	153 (0.4)	40 (0.9)	37 (1.4)	40 (3.5)
PICU admissions, No. (% of RSV hospitalizations)	54 (5.8)	17 (11.0)	<6	<6	6 (15.0)
Adjusted hospital admission rate per 1000 PY (95% CI)^c^	2.24 (2.09-2.38)	3.65 (3.07-4.23)	7.73 (5.27-10.2)	11.55 (7.84-15.27)	28.97 (19.81-38.13)

Abbreviations: LRI, lower respiratory tract infection; PICU, pediatric intensive care unit; PY, person-years; RSV, respiratory syncytial virus.

^a^ There was 1 individual missing data or undetermined on sex.

^b^ There were 4 individuals missing data or undetermined on sex.

^c^ RSV hospitalization rates were estimated using a Poisson regression model with generalized estimating equations to account for repeated measures in children, reported per 1000 PY with 95% CIs. To estimate the influence of prematurity independent from chronic medical conditions, 3 models were created: crude model: only prematurity as the independent variable; adjusted model: sex and presence of chronic medical conditions as additional covariates; and restricted model: limited to children without chronic medical conditions and only prematurity as an independent variable. Only the results of the adjusted model are shown because the results from the other 2 models were similar.

In season-stratified analysis, children born at less than 28 weeks’ GA showed the highest RSV hospitalization rates in their second season, whereas rates were higher in the first season in all other GA groups (Table). Rates in second-season children born at less than 28 weeks’ GA were higher than first-season children overall (RSV hospitalization rate, 8.47 [95% CI, 8.19-8.75] per 1000 person-years), as previously reported in the same population.^5^

## Discussion

This study provides population-level estimates stratified by GA, with season-specific analyses that inform mAb prevention strategies for the most premature infants in a policy-relevant manner. Infants born at less than 28 weeks’ gestation exhibited a persistently elevated risk of RSV hospitalization during their second season, even in the absence of comorbidities. These findings support extending eligibility for long-acting RSV mAbs to infants born at less than 28 weeks’ gestation during their second RSV season. The low first-season hospitalization rates observed in this group likely reflect prolonged neonatal intensive care unit admissions, which limit exposure to respiratory viruses, as well as receipt of palivizumab prophylaxis under historical BC eligibility criteria. In contrast, the elevated second-season risk may reflect long-term alterations in lung development and/or delayed acquisition of RSV-specific immunity. Generalizability beyond BC may be limited by regional differences in RSV epidemiology, health care delivery, and immunization practices, in addition to potential residual confounding. Nonetheless, as new RSV interventions are approved, timely adaptation of prevention guidelines remains critical to ensure optimal protection for infants at highest risk.

## Notes

Supplement 1. **eFigure.** Age and season-based follow-up structure

Supplement 2.
Data Sharing Statement
