# Supplementary material for: RSV Antibody Prophylaxis Needs for Extremely Preterm Infants in Their Second RSV Season
Source: JAMA Pediatr. 2026 Mar 9;180(5):575–7. doi: 10.1001/jamapediatrics.2026.0035 (PMC12973213; doi:10.1001/jamapediatrics.2026.0035)
Supplement: Supplement 1. — eFigure. Age and season-based follow-up structure [file jamapediatr-e260035-s001.pdf]

Supplemental Online Content

Viñeta Paramo M, Watts A, Solimano A, et al. RSV antibody prophylaxis needs for extremely preterm infants in their second RSV season. *JAMA Pediatr*. Published online March 9, 2026.

doi:10.1001/jamapediatrics.2026.0035

eFigure. Age and season-based follow-up structure

This supplemental material has been provided by the authors to give readers additional information about their work.

© 2026 Viñeta Paramo M et al. *JAMA Pediatrics*.

eFigure. Age and season-based follow-up structure

This figure illustrates the key differences between an age-stratified follow-up design (**A**) and a season-stratified follow-up design (**B**). The present study used a season-stratified follow-up, in which outcomes were measured through the end of each child's RSV exposure season.

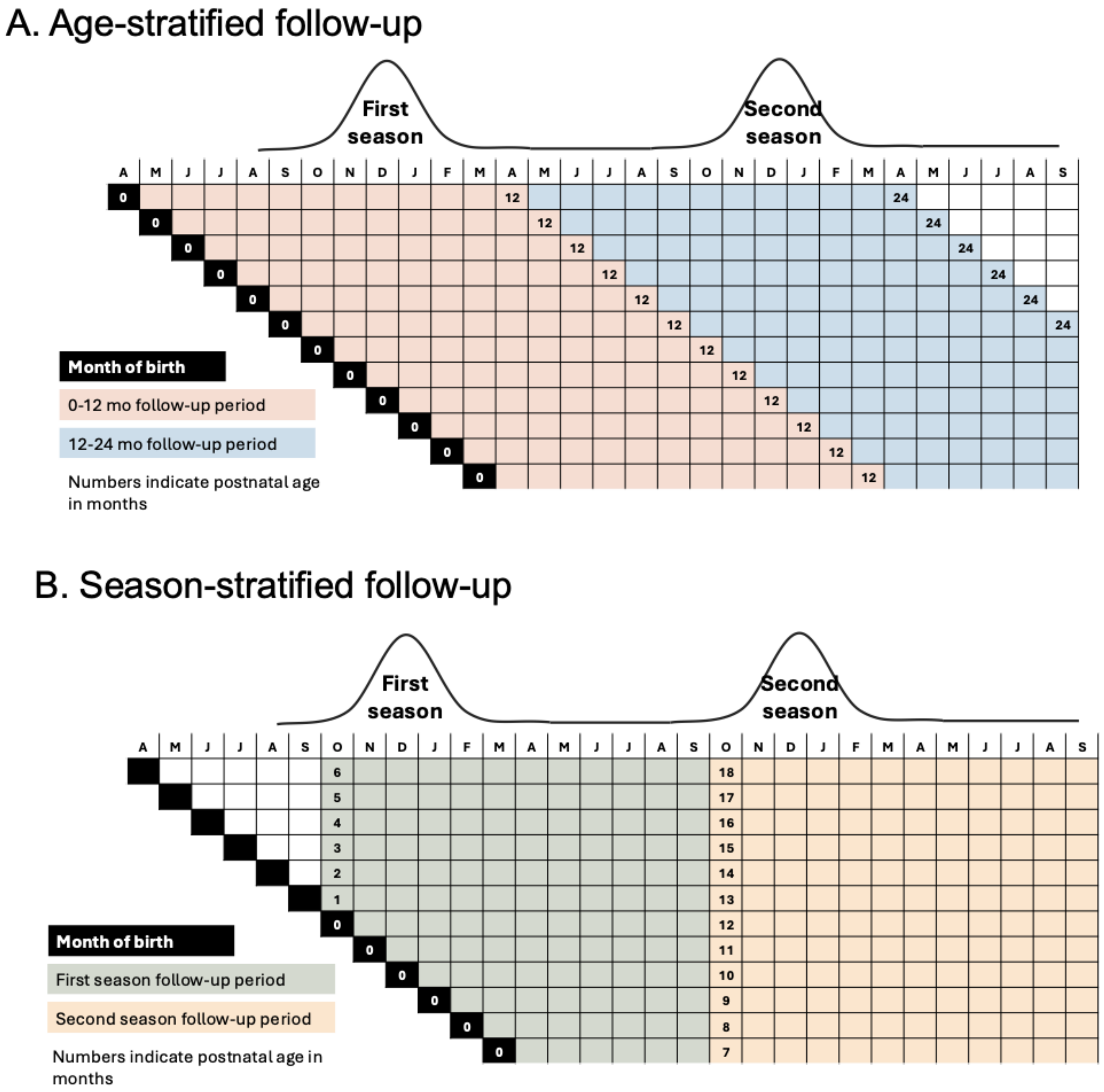

© 2026 Viñeta Paramo M et al. *JAMA Pediatrics*.
