# Supplementary material for: RSV Antibody Prophylaxis Needs for Extremely Preterm Infants in Their Second RSV Season
Source: JAMA Pediatr. 2026 Mar 9;180(5):575–7. doi: 10.1001/jamapediatrics.2026.0035 (PMC12973213; doi:10.1001/jamapediatrics.2026.0035)
Supplement: Supplement 2. — Data Sharing Statement [file jamapediatr-e260035-s002.pdf]

Data Sharing Statement

Viñeta Paramo. RSV Antibody Prophylaxis Needs for Extremely Preterm Infants in Their Second RSV Season. *JAMA Pediatr*. Published March 09, 2026.
doi:10.1001/jamapediatrics.2026.0035

Data
Data available: Yes
Data types: Data dictionary
How to access data: plavoie@bcchr.ca
When available: With publication

Supporting Documents
Document types: Statistical/analytic code
How to access documents: plavoie@bcchr.ca
When available: With publication

Additional Information
Who can access the data: The authors will share the data dictionary and/or code with anyone who requests it, upon request. Access to line-level de-identified data provided by the Data Stewards is subject to approval but can be requested for research projects through the Data Stewards or their designated service providers.
Types of analyses: The data will be made available for any scientifically valid analyses, including secondary analyses, meta-analyses, and methodological studies, subject to approval by the Data Steward, appropriate data-sharing agreements and ethics approval.
Mechanisms of data availability: To make a request for data please visit www.popdata.bc.ca.
